# Non-Covalent Functionalization of Graphene Oxide with POSS to Improve the Mechanical Properties of Epoxy Composites

**DOI:** 10.3390/polym15244726

**Published:** 2023-12-16

**Authors:** Ting Xu, Yumin Jiao, Zhenglian Su, Qin Yin, Lizhou An, Yefa Tan

**Affiliations:** 1 College of Field Engineering, Army Engineering University of PLA, Nanjing 210007, China; xuting@aeu.edu.cn (T.X.); 13951653165@139.com (Z.S.); 13979655839@139.com (L.A.); tanyefa5429@163.com (Y.T.); 2 94789 Troop of PLA, Nanjing 210018, China; 13951687239@139.com

**Keywords:** polyhedral oligomeric silsesquioxane, graphene oxide, non-covalent functionalization, epoxy resin, mechanical properties

## Abstract

Phenyl polyhedral oligomeric silsesquioxane (POSS) is modified onto the GO surface by using the strong π–π coupling between a large number of benzene rings at the end of the phenyl POSS structure and the graphite structure in the GO sheet, realizing the non-covalent functionalization of GO (POSS-GO). The POSS-GO-reinforced EP (POSS-GO/EP) composite material is prepared using the casting molding process. The surface morphology of GO before and after modification and its peel dispersion in EP are examined. Furthermore, the mechanical properties, cross-sectional morphology, and reinforcement mechanism of POSS-GO/EP are thoroughly examined. The results show that the cage-like skeleton structure of POSS is embedded between the GO layers, increasing the spacing between the GO layers and leading to a steric hindrance effect, which effectively prevents their stacking and aggregation and improves the dispersion performance of GO. In particular, the 0.4 phr POSS-GO/EP sample shows the best mechanical properties. This is because, on the one hand, POSS-GO is uniformly dispersed in the EP matrix, which can more efficiently induce crack deflection and bifurcation and can also cause certain plastic deformations in the EP matrix. On the other hand, the POSS-GO/EP fracture cross-section with a stepped morphology of interlaced “canine teeth” shape is rougher and more uneven, leading to more complex crack propagation paths and greater energy consumption. Moreover, the mechanical meshing effect between the rough POSS-GO surface and the EP matrix is stronger, which is conducive to the transfer of interfacial stress and the strengthening and toughening effects of POSS-GO.

## 1. Introduction

Owing to its excellent physical, chemical, and mechanical properties, including good wear and corrosion resistance, chemical stability, high bonding strength, low shrinkage rate, low cost, and easy processing and molding, epoxy resin (EP) is widely used in the preparation of adhesives, composite materials, and coatings in different fields such as chemical engineering, aerospace, and electronic information [1,2,3,4]; moreover, it is currently one of the most popular thermosetting resins in the world. However, the high cross-linking density of EP after curing can cause significant internal stress in the three-dimensional structure, reducing the mechanical strength, impact toughness, conductivity, and thermal conductivity of the composite material, thereby limiting its extensive application in high-performance engineering fields [5,6]. Therefore, further strengthening and toughening treatments of EPs have received considerable research attention.

Over the recent years, nanomaterials have been widely used to enhance the mechanical properties, durability, friction resistance, and overall performance of EP composites.

For example, Amirbek et al. [7] used amino acetic acid-functionalized AlN to strengthen epoxy resin. The results show that the functionalized AlN particles have good dispersibility and have significant effects on the structure, curing kinetics, and physicochemical and mechanical properties of epoxy nanocomposites. The addition of 0.5 wt% amino acetic acid AlN particles can achieve the maximum strengthening effect. Asad et al. [8] used pristine-, COOH-, and NH_2_-functionalized CNTs as reinforcements to enhance epoxy resin. The results show that varying the weight fraction and changing the type of CNTs caused an increment in the Young’s modulus, tensile strength, and thermal stability of the nanocomposites; moreover, the nanocomposites containing 0.5 wt% NH_2_-functionalized CNTs offer the best combination of thermal and mechanical properties. Hesam et al. [9] prepared aminopropyl trimethoxysilane-functionalized graphene (SGr) and studied the effects of adding various amounts of SGr on the mechanical properties and wear behavior of epoxy nanocomposites. The outcome suggested that the tensile, compressive, ILSS, and wear properties of SGr-epoxy samples were enhanced. The above research focused on solving the problem of easy aggregation of nanomaterials and showing that nanomaterial enhancement and surface functionalization of fillers are very effective modification methods that can significantly enhance polymer nanocomposites. However, the enhancement effect of nanofillers on different mechanical properties does not seem to have a uniform law, and the related influence law needs to be further studied and verified.

**Figure 1:**
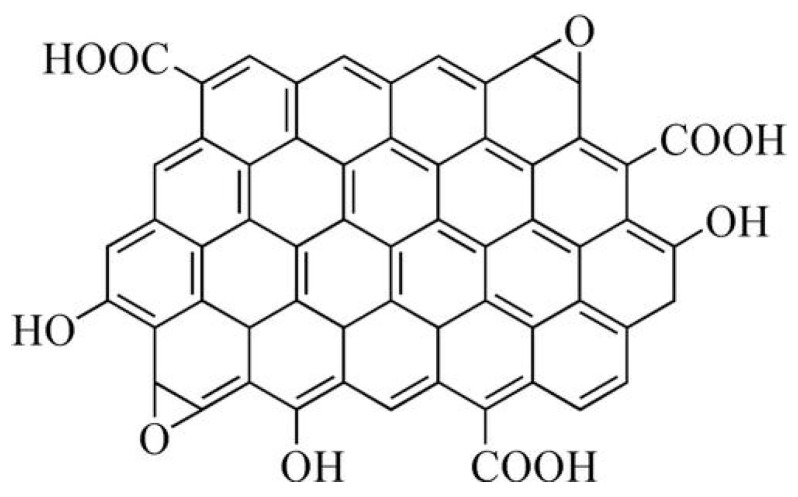
Molecular structure of graphene oxide (GO).

Graphene oxide (GO) is a quasi-two-dimensional lamellar nanomaterial whose molecular structure is shown in Figure 1. Owing to its unique two-dimensional honeycomb lattice structure with extended π bond conjugation, GO exhibits excellent mechanical, thermal, and electrical properties, and it can significantly improve the toughness, thermal resistance, and mechanical properties of EPs [10,11]. However, due to the strong hydrogen bonding and the dipole force of oxygen-containing groups between the GO layers, irreversible aggregation is prone to occur, seriously affecting its dispersion performance in EPs. Moreover, the weak interfacial bonding between GO and EP greatly limits the engineering application of GO-reinforced EP composites [12].

To alleviate the poor dispersion and easy agglomeration of GO in EP, the current modification methods mainly include: taking the oxygen-containing group on the surface of GO as the active site; and grafting organic molecules on the surface of GO through a covalent functionalization reaction to improve the dispersion and interface compatibility of GO in the resin or modify GO through non-covalent interactions, such as π–π interactions, hydrogen bonding, or van der Waals forces, to promote its exfoliation and dispersion in the resin. For example, Wan et al. [13] synthesized epoxy composites with silane-functionalized GO at different loadings by grafting silane containing epoxy ended-groups onto the surface of GO sheets. The results showed that the interfacial adhesion between GO and EP was effectively improved by the covalent grafting layer, and the tensile strength, bending strength, and fracture toughness of the composite were significantly improved compared with those of pure EP. Li et al. [14] functionalized GO with p-phenylenediamine (GO-PPD) to prepare GO-PPD/CE-EP composites with a blend of cyanate ester (CE) and EP as the matrix. The results suggested that the introduction of GO-PPD reduced the curing temperature of the resin and significantly improved its mechanical and thermal properties. Ding et al. [15] grafted GO with a silane coupling agent to synthesize functionalized reduced GO (F-RGO) and then prepared functionalized reduced GO-reinforced bisphenol A cyanate ester (F-RGO/CE) nanocomposites using a solution intercalation method. It was observed that the impact strength, bending strength, tensile strength, Young’s modulus, and elongation at break of the composite material with the optimal content (3 wt.% F-RGO nanosheets) were significantly improved compared with those of pure CE. Although methods such as covalent functionalization and GO modification are indeed effective, the complex synthesis methods, chemical treatment, and cumbersome process flow limit their wide application and may even create additional defects on the surface of GO, thereby damaging its main structure. Therefore, to simplify the synthesis method and optimize the utilization of the inherent structure and mechanical properties of GO, the non-covalent functionalization of GO is a more effective modification method [16]. Additionally, it is particularly critical to choose the right non-covalent modifier.

**Figure 2:**
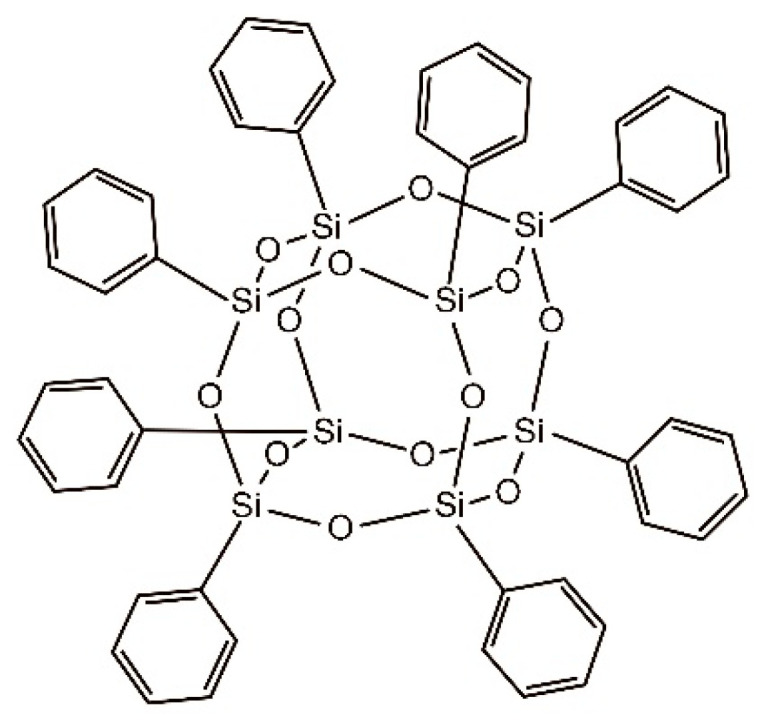
Molecular structure of polyhedral octameric silsesquioxane (POSS) (Phenyl POSS).

Polyhedral oligomeric silsesquioxane (POSS) is a new form of nano-silica with the empirical formula (RSiO_1.5_)_8_, where R is a hydrogen atom or an organic functional group such as an alkyl, alkylene, acrylate, hydroxyl, or epoxide unit [17,18]. A typical molecular structure is shown in Figure 2. POSS has a unique, highly symmetrical, cubic cage-like skeleton structure with a diameter of only 1–3 nm, and it exhibits good dispersion, low density, and high thermal stability. Its inorganic core endows it with good dielectric properties, optical properties, thermal stability, mechanical properties, and flame retardancy. Through blending, grafting, cross-linking, and copolymerization, POSS can be doped in almost all polymer matrices to enhance various properties of polymers at the molecular level, such as thermal stability, corrosion resistance, tensile strength, modulus, impact toughness, etc. For instance, Jiang et al. [19] successfully grafted monofunctional (methacrylolsobutyl) and multifunctional (methacryl) POSS onto the surface of carbon fibers (CFs) to improve the interfacial strength of CF-reinforced unsaturated polyester resin (UPR) composites. The results revealed that compared with the unmodified CF/UPR composite, the interlayer shear strength, interfacial shear strength (IFSS), and impact energy of the methacryl POSS-grafted CF/UPR composite increased by 42.6%, 102.2%, and 72%, respectively. Zhao and Huang [20] grafted a uniform layer of octaglycidyldimethylsilyl POSS onto the surface of CFs to improve the interfacial properties between the CFs and the EP matrix. The results showed that the grafting of POSS significantly increased the surface roughness, oxygen-containing functional groups, and fiber surface energy of CFs. Compared with the unmodified composites, the interlaminar shear strength (ILSS) of the modified composites increased from 68.8 MPa to 90.5 MPa, and the impact toughness increased from 2.62 J to 3.59 J. Liu [21] used the synthesized POSS as a graft agent to covalently react with GO to make POSS-G-GO, prepared EP/POSS-G-Go nanocomposites, and verified the success of modification through FTIR, XRD, DMA, and TGA analyses. The results showed that the tensile strength, flexural strength, impact strength, and elastic modulus of the composite were significantly improved compared with pure EP when the addition of POSS-g-GO was 2 wt%. Therefore, POSS exhibits immense application potential in the functionalization of GO. However, the dispersion of POSS non-covalent functionalized GO in EP and its enhancement effect on the mechanical properties of epoxy composite materials have been rarely examined.

In this study, to address the issues of poor dispersion and easy agglomeration of GO in EP, a convenient method is proposed to synthesize EP composites. Specifically, phenyl POSS (as shown in Figure 2) is taken as the modifier, and the strong π–π coupling between a large number of benzene rings at the end of the phenyl POSS structure and the graphite structure in GO lamella are used to modify phenyl POSS onto the GO surface, thereby realizing the non-covalent functionalization of GO. Finally, POSS-coated GO (POSS-GO) nano-hybrid tablets are synthesized, and GO and POSS-GO-reinforced epoxy composites (POSS-GO/EP) are prepared using the solution casting method. The POSS-GO and POSS-GO/EP samples are characterized by several techniques, including Fourier transform infrared (FTIR) spectroscopy, X-ray photoelectron spectroscopy (XPS), Raman spectra, thermogravimetric analysis (TGA), X-ray diffraction (XRD), dynamic light scattering (DLS), scanning electron microscopy (SEM), and transmission electron microscopy (TEM). Moreover, the effects of POSS-GO on the mechanical properties of the EP composite, such as the microhardness, fracture toughness, tensile strength, and Young’s modulus, are comprehensively examined. Finally, the strengthening and toughening mechanism of POSS-GO on EP is revealed to provide technical support for boosting the engineering application of POSS-GO in epoxy composites.

## 2. Experiment

### 2.1. Raw Materials

GO with a sheet diameter of approximately 2.6 mm and a purity of more than 95% was obtained from Nanjing Jicang Nano Technology Co., Ltd. (Nanjing, China). Phenyl POSS, N, N-dimethylacetamide (DMAc), and ethanol were purchased from Shanghai Aladdin Biochemical Technology Co., Ltd. (Shanghai, China). IN2 infusion EP and AT300SLOW curing agent (IN2:AT300SLOW = 100:30) were obtained from Beijing Composite Yigou Technology Co., Ltd. (Beijing, China).

### 2.2. Preparation and Mechanism of Phenyl POSS-Functionalized GO (POSS-GO)

**Figure 3:**
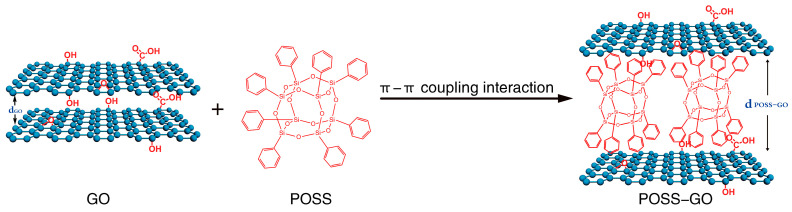
Schematic for the preparation principle of phenyl POSS-functionalized graphene oxide (POSS-GO).

(1) Firstly, 100 mg of GO was weighed and then dried at 80 °C for 2. Subsequently, it was dissolved in 100 mL of DMAc dispersion at room temperature through mechanical stirring (model XFK JJ-1A, stirring speed: 300 rpm) and an ultrasonic water bath treatment (model JP-010S) for 15 min.
(2) The GO dispersion was transferred to a 500 mL three-necked flask, and then 100 mL of DMAc and 50 mg of POSS were added to it. The solution was mechanically stirred at room temperature (300 rpm) and simultaneously treated with an ultrasonic water bath for 2 h.
(3) The solution was filtered under reduced pressure using a polytetrafluoroethylene (PTFE) filter membrane with a pore size of 0.45 µm, and the filtered material was washed 5 times with anhydrous ethanol. Then, the solvent was placed in a vacuum oven and dried under vacuum at 80 °C for 12 h. Finally, the phenyl POSS non-covalent functionalized GO (abbreviated as POSS-GO) was obtained. The preparation principle is shown in Figure 3.

### 2.3. Preparation of POSS-GO-Reinforced EP (POSS-GO/EP) Composite Material

**Figure 4:**
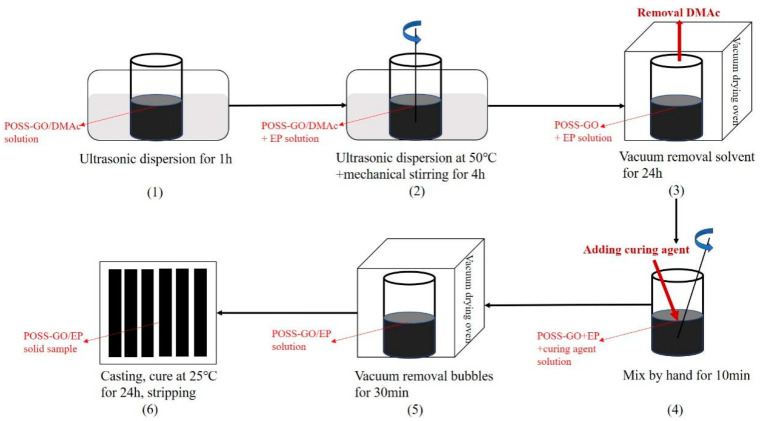
Preparation process of POSS-GO/EP composite materials.

The POSS-GO-reinforced EP (POSS-GO/EP) composite samples were prepared using the pouring method, and the specific steps are shown in Figure 4.

(1) An appropriate amount of POSS-GO was dispersed in a DMAc solution, which was then sonicated in a water bath for 1 h to obtain the POSS-GO/DMAc dispersion.
(2) The POSS-GO/DMAc dispersion was injected into an EP solution, and the mixture was subjected to an ultrasonic water bath treatment at 50 °C for 4 h. At the same time, a high-speed mechanical mixer was used to stir the mixture at 500 rpm.
(3) The dispersed mixture was placed in a vacuum dryer, and the solvent DMAc removal was realized through a vacuum pump for 24 h.
(4) The mixture with DMAc removed was mixed with the curing agent at a ratio of 100:30 and stirred for 10 min to allow for them to be completely well-mixed.
(5) The fully mixed mixture was placed in a 25 °C constant-temperature vacuum chamber for 30 min to eliminate bubbles.
(6) Finally, the mixture was casted into a PTFE mold, cured at 25 °C for 24 h, and demolded to prepare the POSS-GO-reinforced EP (POSS-GO/EP) composite material.

In addition, to explore the reinforcement effect and mechanism of POSS-GO/EP, non-functionalized GO-reinforced EP (GO/EP) composite materials were prepared as comparison samples, and the preparation process was the same as above. The specific composition of various types of samples is shown in Table 1. The phr in Table 1 is the abbreviation of parts per hundred parts of resin, which represents the amount of substance to be added with each 100 parts of resin. For example, 50 phr represents 100 g of resin, meaning that 50 g of the substance need to be added.

### 2.4. Characterization

The functional groups on the surface of GO and POSS-GO samples were analyzed using FTIR spectroscopy (German Bruker Manufacturing Company, Luken, Germany, Model Tensor 27). The test sample was prepared using a potassium bromide pressed-disk technique, and a total of 32 scans were conducted with a scanning resolution of 4 cm^−1^ and scanning range of 400–4000 cm^−1^. The sample needed to be dried for 24 h before testing to remove moisture. The chemical compositions and functional groups of GO and POSS-GO were analyzed by a photoelectron spectrometer (XPS) (ESCALAB 250XI, Waltham, MA, USA). An AlKα X-ray light source was used in the test. The vacuum of the sample chamber was 2 × 10^−8^ Pa, and the excitation power and voltage were 40 W and 15 KV, respectively. Raman spectroscopy (Aramis, Horiba Jobin Yvon, Paris, France) was used to measure the Raman spectra of GO and POSS-GO; the excitation source was a 532 nm He-Ne laser line, and a 50× objective lens was used to focus the laser beam on the sample surface (laser point: 200 μm). The thermal stability of GO and POSS-GO samples was examined using TGA (209 F3 Tarsus, Selb, Germany), and the adsorption amount of POSS on GO was quantified. The experiment was conducted in a nitrogen atmosphere with a heating rate of 10 °C/min and a maximum heating temperature of 800 °C. The crystal structure of GO and POSS-GO samples was analyzed using XRD (D8 Advance, Bruker) with a Cu Kα Target (λ = 0.154178 nm) under an operating voltage of 40 KV, operating current of 200 mA, scanning rate of 8°/min, and scanning range of 0–50°. Based on a dynamic light scattering technique, the particle size and distribution of GO and POSS-GO were accurately measured by a Zeta Sizer 3000 (Malvern Instruments, Malvern UK) dynamic light scattering particle size analyzer. The test adopts a He-Ne light source with a power of 10 mW, wavelength of 633 nm, scattering angle of 90°, and temperature of 25 °C. The cross-sectional micromorphology of the pure EP, GO/EP, and POSS-GO/EP tensile samples was examined using SEM (Hitachi S-4800, Hitachi, Tokyo, Japan), where the acceleration voltage was 15 KV. Before the observation, the samples were coated with gold using an ion-sputtering instrument for 2 min. The microstructure and dispersion state of GO and POSS-GO were investigated using high-resolution TEM (Hitachi HT-7800, Hitachi, Japan). For the TEM analysis, the samples needed to be cut into very thin slices using an ultramicrotome, where the slice size was approximately 1 mm × 1 mm and the slice thickness was nearly 50–60 nm. The slices were collected in a water tank and were deposited on the surface of a 200-mesh copper net.

**Figure 5:**
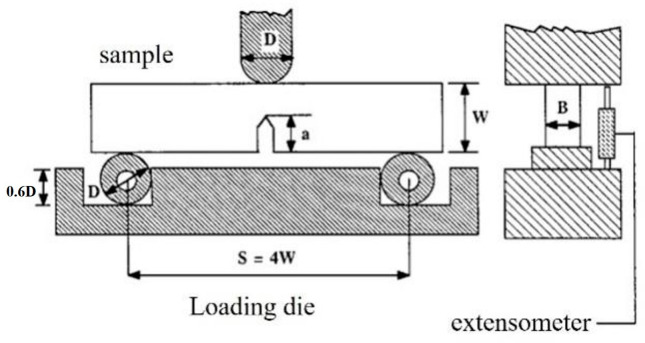
Schematic for the fracture toughness test of composite materials.

The microhardness of pure EP, GO/EP, and POSS-GO/EP samples was measured using a D-type Shore Durometer hardness tester (SHYC445D, Laizhou Huayin Testing Instrument Co., Ltd., Laizhou, China). The samples’ fracture toughness was tested using the electro-hydraulic servo fatigue testing machine (PWD-10S type, Jinan Zhongluchang Testing Machine Manufacturing Co., Ltd., Jinan, China) according to the ASTM D5045-99 standard. A cuboid three-point bending sample with a single-edge notched beam (SENB) was used, where the sample size was 50 mm × 10 mm × 5 mm (see Figure 5). A prefabricated crack was introduced into the cuboid sample using a sharp blade. The experiment was carried out at room temperature under a loading rate of 1 mm/min, and 5 samples were tested for each group. The fracture toughness (*K_IC_*) and critical energy release rate (*G_IC_*) were calculated as follows [22]:

(1) $$K_{IC}=G\frac{P_{Q}S}{BW^{3/2}}$$

(2) $$G_{IC}=\frac{1-\nu^{2}}{E}K_{IC}^{2}$$

where *G* is the crack shape factor, *P_Q_* is the load of conditional crack instability propagation, *S* is the span, *B* is the sample thickness, *W* is the sample width, and *E* is the elastic modulus of the sample. The Poisson’s ratio *v* of EP and its composite materials is 0.35 [23].

**Figure 6:**
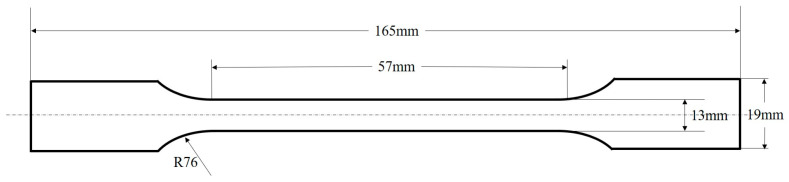
Size diagram of the tensile specimens of composite materials.

The tensile strength and Young’s modulus of pure EP, GO/EP, and POSS-GO/EP samples were measured using a microcomputer-controlled electronic universal testing machine (DN-W5KN type, Zhejiang Dana Automation Technology Co., Ltd., Zhejiang, China) in accordance with the ASTM D 638-2010 standard [24]. The samples were dumbbell shaped, and their dimensions are shown in Figure 6. The experiment was conducted at room temperature with a loading rate of 5 mm/min, and 5 samples were tested for each group.

## 3. Results and Discussion

### 3.1. Characterization of Non-Covalent Functionalized GO

**Figure 7:**
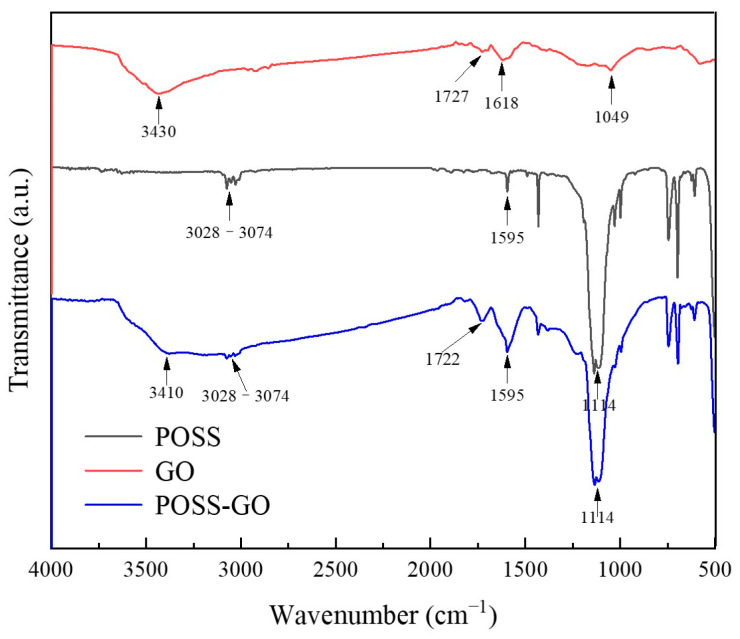
FTIR spectra of GO, POSS, and POSS-GO nanosheets.

Figure 7 shows the FTIR spectra of GO, POSS, and POSS-GO nanosheets. In the spectrum of GO nanosheets, the absorption peaks at 3430 and 1727 cm^−1^ correspond to the stretching vibrations of O-H on carboxylic acid and hydroxyl group [25] and C=O on carboxylic acid [26], respectively; the absorption peak at 1049 cm^−1^ indicates the presence of C-O-C groups on GO [27]. In addition, the presence of conjugated structures (C=C, 1618 cm^−1^) on the surface of GO can be observed. In the FTIR spectrum of POSS, the absorption peak at 1114 cm^−1^ is attributed to the stretching vibration of Si-O-Si [28]. The absorption peak at 3028–3074 cm^−1^ represents methylene group (-CH2-), and the peak at 1595 cm^−1^ corresponds to the stretching vibration of the conjugated C=C group on the benzene ring. In the FTIR spectra of POSS-GO, the peaks at 3028–3074 cm^−1^ and 1114 cm^−1^ are attributed to the stretching vibrations of methylene (-CH2-) and Si-O-Si groups from POSS, respectively; the peak at 1595 cm^−1^ represents the stretching vibration of the conjugated C=C group on the benzene ring. At the same time, it can be clearly seen that this peak is sharper than POSS, which is due to the effect of the superposition of C=C in GO and C=C in POSS here. Comparing the FTIR spectra of POSS-GO and POSS, an absorption peak at 3410 cm^−1^ appears in the spectrum of POSS-GO, which comes from the stretching vibration of O-H on the carboxylic acid and hydroxyl group in GO. In addition, an absorption peak at 1722 cm^−1^ can be observed in the spectrum of POSS-GO, which is the stretching vibration peak of the C=O group on carboxylic acid in GO. The presence of these peaks in POSS-GO proves that POSS has been successfully modified onto the GO nanosheets.

**Figure 8:**
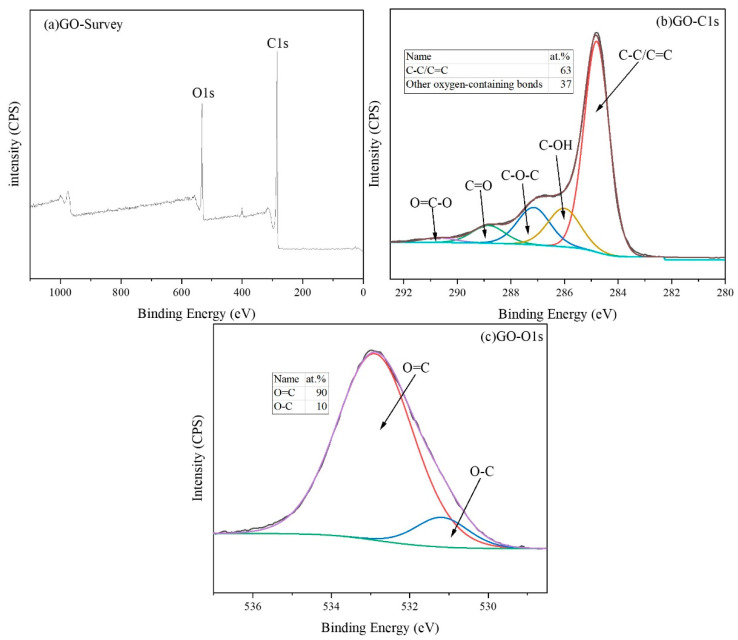
XPS spectrum of GO (**a**) full spectrum; (**b**) C1s spectrum; (**c**) O1s spectrum.

**Figure 9:**
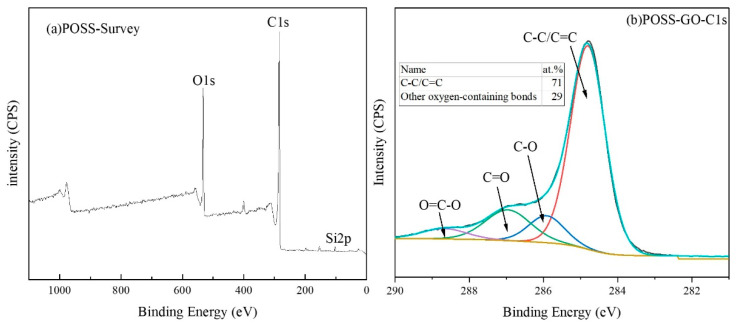

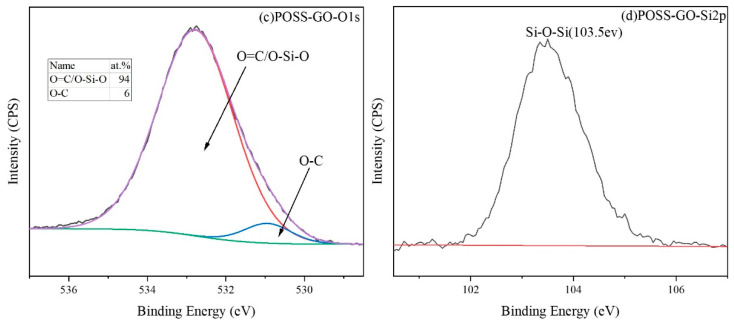
XPS spectrum of POSS-GO (**a**) full spectrum; (**b**) C1s spectrum; (**c**) O1s spectrum; (**d**) Si2p spectrum.

In order to further investigate the changes in the chemical composition and functional groups of GO before and after functionalization and confirm the effectiveness of the functionalization treatment, XPS tests were conducted on GO and POSS-GO, and the test results are shown in Figure 8 and Figure 9. As can be seen from the full spectrum of GO in Figure 8a, C and O are the main constituent elements of GO. Figure 8b shows the C1s spectrum of GO. It can be seen that at 284.8 ev, 286 ev, 287 ev, 288.8 ev, and 290.5 ev, the corresponding functional groups are C=C/C-C, C-OH, C-O-C, C=O, and O-C=O, respectively, which is consistent with previous reports [29]. Figure 8c shows the O1s spectrum of GO, which shows two valence states, namely O=C (532.9 ev) and O-C (531.2 ev).

After POSS functionalization, the Si element was added to the POSS-GO full spectrum in Figure 9a. Meanwhile, from the C1s spectrum of POSS-GO in Figure 9b, it can be seen that the C=C/C-C in POSS-GO increases compared with GO, thanks to the introduction of the benzene ring in POSS. In addition, as can be seen from the O1s spectrum of POSS-GO in Figure 9c, the peak area proportion at 532.9 ev is increased compared with GO, which is due to the introduction of O-Si-O bond in POSS. Figure 9d shows the Si2p spectrum of POSS-GO. The binding energy difference between Si2p_1/2_ and Si2p_3/2_ is about 0.5 ev, which is much smaller than the resolution of conventional XPS (~1 ev). Conventional XPS can only obtain one peak, which is usually the average of 2p_1/2_ and 2p_3/2_, and can be used to represent the chemical valence of Si without the need for peak splitting [30]. The Si2p spectrum of POSS-GO further confirms the introduction of O-Si-O bonds on the GO surface. In summary, the above results fully demonstrate that POSS has been successfully modified onto the GO layer.

Raman spectroscopy can reflect the degree of surface defects and the quality of the crystal structure of carbon nanofillers, and it can be used to investigate the effect of chemical reactions on the structure of carbon nanofillers during preparation. For carbon materials, the D-mode located near 1340 cm^−1^ is caused by the defect of the sp3 hybrid carbon structure; the G-mode located near 1570 cm^−1^ is usually caused by the in-plane bonding stretching motion of the sp2 hybrid carbon structure atomic pairs. Therefore, the strength ratio of D-mode to G-mode (I_D_/I_G_) can be used to characterize the defect density of carbon materials. The higher the I_D_/I_G_ value, the greater the GO defect density and the greater the degree of damage to the GO structure caused by the functionalization process.

**Figure 10:**
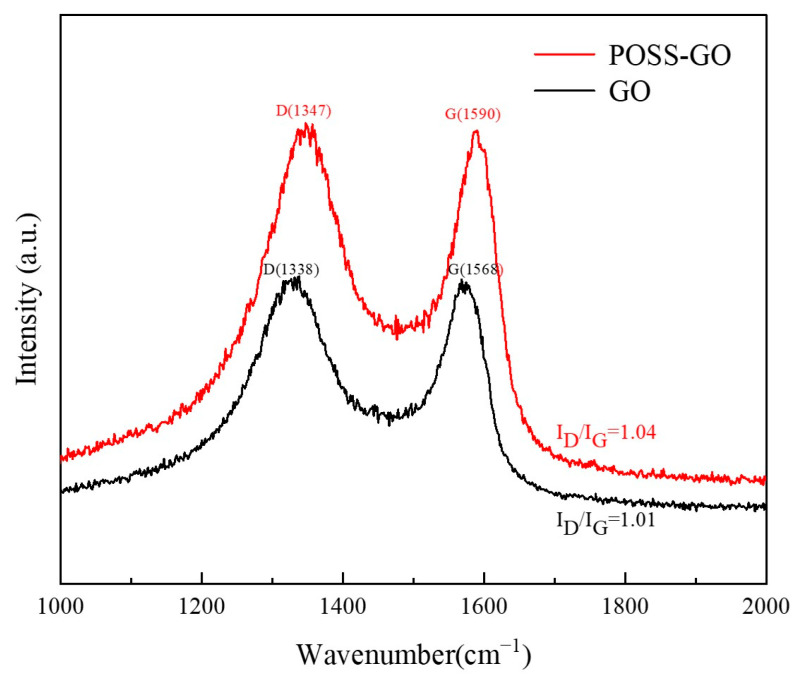
Raman spectra of GO and POSS-GO.

Figure 10 shows the Raman spectra of GO and POSS-GO. As can be seen from Figure 10, the defect mode (D mode) and tangential vibration mode (G mode) of GO appear at 1338 cm^−1^ and 1568 cm^−1^, respectively. Compared with GO, the D-mode and G-mode of POSS-GO have increased by 9 cm^−1^ and 22 cm^−1^, respectively. The upward shift of D- and G-mode indicates a strong intermolecular interaction between POSS and GO, namely the π–π stacking interaction between the benzene ring on POSS and the side walls of the GO graphite structure. Previous studies have also reported the phenomenon of D-mode and G-mode upshift caused by π–π stacking interactions [31]. Meanwhile, according to calculations, the I_D_/I_G_ values of GO and POSS-GO are 1.01 and 1.04, respectively, with a very small difference. This indicates that the non-covalent functionalization method used in this paper introduces fewer defects into GO and effectively preserves the inherent layered structure of GO.

**Figure 11:**
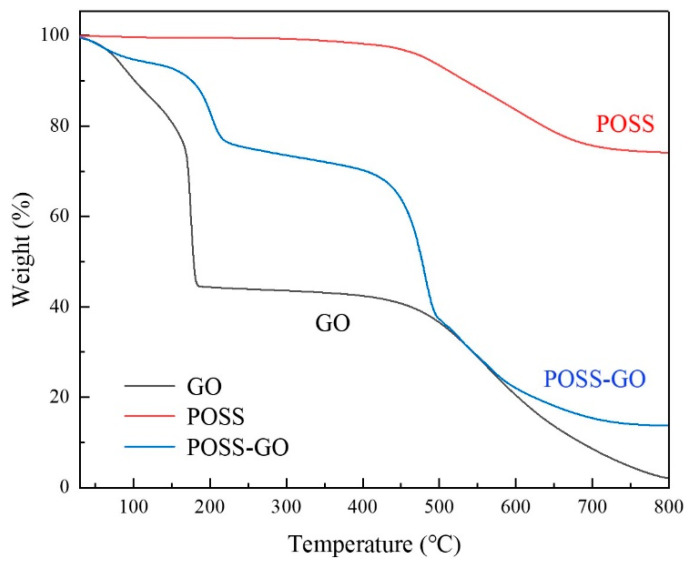
TGA curves of GO, POSS, and POSS-GO samples.

Figure 11 shows the TGA curves of GO, POSS, and POSS-GO samples. In the TGA curve of GO, the weight loss below 150 °C is caused by the evaporation of water adsorbed on the surface of GO. GO undergoes intense decomposition within the range of 150–250 °C, which is due to the decomposition of a large number of unstable oxygen-containing functional groups (-OH- and -COOH-) on the GO layer. Meanwhile, stable mass loss can be observed in the range of 420–700 °C, which is caused by the decomposition of the GO carbon skeleton [32]. The final residual mass is approximately 2.07 wt.%, which suggests a total weight loss rate of 97.93%. The TGA spectrum of POSS indicates a good thermal stability, with only a 2% thermal weight loss in the range of 0–400 °C. It exhibits stable mass loss in the range of 400–700 °C, which is attributed to the condensation of the Si-O skeleton and the decomposition of the benzene ring carbon structure in POSS [33]. The final residual mass is nearly 74.07 wt.%, which implies a total weight loss rate of 25.93%. According to the TGA curve of POSS-GO, under the same temperature, the thermal weight loss of POSS-GO is smaller than that of GO, indicating that the thermal stability of POSS-GO is better than that of GO. In the TGA curve of POSS-GO, there are two obvious weight loss ranges. The weight loss occurring within the temperature range of 120–250 °C is mainly caused by the decomposition of GO, while that occurring in the range of 450–700 °C is primarily caused by the decomposition of POSS adsorbed on GO. The final residual mass is approximately 13.78 wt.%, which implies a total weight loss rate of 86.22%. According to the final residual mass of different samples at 800 °C (POSS: 74.07%; GO: 2.07%; and POSS-GO: 13.78%), it can be inferred that the adsorbing amount of POSS on GO nanosheets is 16.7%.

**Figure 12:**
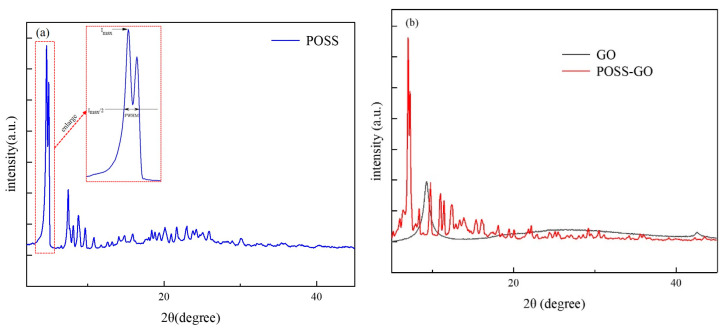
XRD spectra of (**a**) POSS, (**b**) GO, and POSS-GO samples.

The structures of POSS, GO, and POSS-GO samples were analyzed using XRD, and the results are shown in Figure 12. Figure 12a shows the XRD spectrum of POSS. It can be seen that POSS has a strongest diffraction peak at 2θ = 4.6°, and its grain size can be calculated using the Scherrer formula [34] (Formula (3) below):

(3) $$D=\frac{k\lambda }{\beta cos\theta }$$

In Formula (3), k is the Scherrer shape factor, whose value is 0.89; λ is the wavelength of the incident X-ray wave (λ = 0.154178 nm). β is the half-peak width of the diffraction peak, and its value is about 0.1 according to the test results. θ is the Bragg diffraction angle, which has a value of 2.3° according to the test results. Therefore, it can be calculated that the grain size of POSS is about 1.37 nm.

It can be seen from Figure 12b that GO has a strong diffraction peak at 2θ = 9.3°, which shifts to a lower angle of 2θ = 7° in POSS-GO. The interlayer spacing d of GO and POSS-GO can be calculated using the Bragg formula [35] as follows:

2*dsin*θ = *nλ* (4)

where λ is the wavelength of the incident wave (λ = 0.154178 nm) and n is the reflection order, where n is taken as 1.

The interlayer spacing of GO before functionalization is calculated to be 0.95 nm, which increases to 1.28 nm after POSS functionalization (POSS-GO sample).

The above results indicate that the POSS modified on the surface of GO can further stretch the adjacent GO layers to a certain extent, causing the stacked GO to peel off and separate. This can be attributed to the cage-like skeleton structure of POSS, where a large number of benzene rings at the end of this structure act like “tentacles” and firmly adhere to the surface of the GO layer through strong π–π coupling, enabling POSS to be embedded between adjacent GO layers (as shown in Figure 4); this leads to a steric hindrance effect between GO layers, effectively blocking their proximity and stacking and avoiding their agglomeration. At the same time, it can be seen from the XRD spectrum of POSS-GO that its peak is sharper and narrower than that of GO. This may be because the introduction of the new POSS molecular structure on the surface of GO changes the original completely ordered structure between the layers. Theoretically, the regularity of molecular structure can affect its dispersibility in the solvent. The lower the regularity, the easier its dispersion in the solvent. This also provides a theoretical basis for the better organic dispersion performance of POSS-GO in the following research, which will help POSS-GO to give full play to the reinforcement and toughening effect.

### 3.2. Morphology and Dispersion Properties of Non-Covalent Functionalized GO

**Figure 13:**
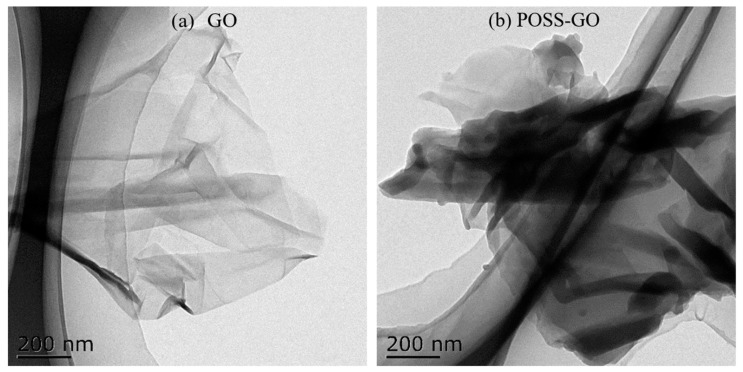
TEM images of GO and POSS-GO nanosheets: (**a**) GO (**b**) POSS-GO.

TEM was used to visualize the morphological characteristics of GO and POSS-GO samples, and the obtained images are shown in Figure 13. It can be seen that GO has a very obvious layered structure, with a relatively clean and smooth surface. Due to its small atomic thickness and thin layer (approximately 3–5 µm in size), GO exhibits a highly transparent surface (Figure 13a). Meanwhile, due to the abundance of oxygen-containing functional groups (-OH-, -COOH-, etc.) on the surface of GO sheets, their interactions are very strong, which can easily lead to the formation of wrinkles on the surface of GO sheets. Therefore, the GO surface has a certain degree of wrinkles, curls, and folding structures. As shown in Figure 13b, the surface of POSS-GO after functionalization treatment becomes significantly rougher, with more significant surface wrinkles and a larger layer thickness compared with GO. Additionally, a layer of black opaque additive is clearly attached to the surface, which is primarily because POSS is grafted onto the surface of GO, weakening the sample’s electron transmission ability. These observations indicate that after the functionalization treatment, POSS successfully adheres to the surface of GO.

In order to further study the dispersion of GO before and after modification, Dynamic light scattering (DLS) tests were carried out on GO and POSS-GO. DLS takes the particle size of nanoparticles and the polymer dispersity index (PDI) as the main evaluation parameters to obtain the distribution state of particle size, which is the statistical data of a large number of particles and has strong persuasiveness. Compared with SEM or TEM, the most prominent advantage of DLS is that it can measure the diameter of the agglomeration of nanoparticles, which can more directly observe and evaluate the agglomeration of nanoparticles.

**Figure 14:**
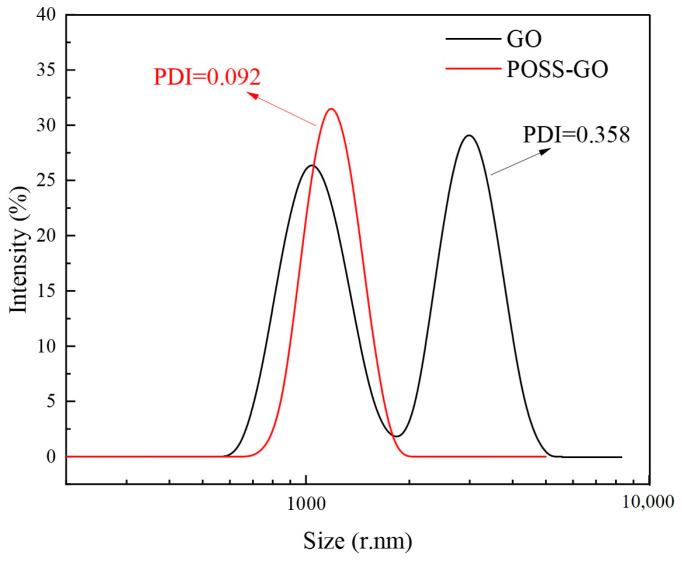
Particle size distribution of GO and POSS-GO measured by dynamic light scattering.

The DLS curves of GO and POSS-GO are shown in Figure 14. As can be seen from Figure 14, the DLS curve of POSS-GO has a single peak, and the cumulative scattered light intensity shows that the particle size is distributed in the range of 860–1600 rnm, which may be the particle size of a single molecule, indicating that it is an intramolecular association product. The DLS curve of GO changes from unimodal to bimodal, with a slightly smaller peak in the particle size distribution range of 740–1500 rnm, indicating the presence of intramolecular association products; and a slightly larger peak in the particle size distribution range of 2000–4400 rnm, which may be the particle size of aggregates produced by intermolecular association, indicating the presence of intermolecular agglomeration products [36]. In particular, POSS-GO has a PDI index of 0.092, and GO has a PDI index of 0.358. PDI is a dimensionless value reflecting the width of the particle size distribution, which characterizes the size dispersion of nanomaterials, ranging from zero to one. The smaller the value, the more uniform the particle size and the better the dispersion [37]. Therefore, the DLS test results further prove that POSS plays a promoting role in improving the dispersion of GO.

**Figure 15:**
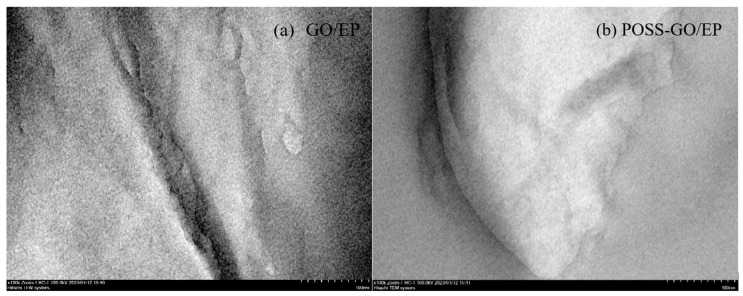
TEM images of GO and POSS-GO samples dispersed in the EP matrix: (**a**) GO/EP; (**b**) POSS-GO/EP.

To further explore the dispersion state of GO and POSS-GO samples in the EP matrix, ultra-thin slices of GO/EP and POSS-GO/EP were visualized using TEM, and the results are shown in Figure 15. In Figure 15a, obvious black areas are observed in the TEM image of GO/EP, which are formed by the serious accumulation of GO in the EP matrix, indicating that the non-functionalized GO has poor dispersion in the EP matrix. According to Figure 15b, after POSS functionalization treatment, POSS-GO is uniformly dispersed in the EP matrix, and no obvious GO aggregates are found, indicating that POSS-GO has good dispersion performance in the EP matrix. This is because phenyl POSS is firmly adsorbed on the surface of GO through the strong π–π coupling between its benzene ring group and GO, which effectively prevents the agglomeration of GO and improves the dispersion state of GO in EP. The dispersion performance of GO in the EP matrix plays a decisive role in its reinforcement and toughening effect on composite materials. If GO has a poor dispersion in EP, internal defects and stress concentration can be formed in the composite material due to the accumulation of GO under stress, resulting in silver lines or cracks, greatly weakening the strengthening and toughening effect of GO. If GO has good dispersibility in EP, it can be uniformly intercalated into EP in a layered structure. When external loads are transferred from the EP matrix to the GO layer, GO can effectively play a skeletal support role, fully leveraging its excellent mechanical performance to strengthen and toughen the composite materials. Therefore, the good dispersion performance of POSS-GO in EP matrix is conducive to enhancing the mechanical properties of POSS-GO/EP composite materials.

### 3.3. Mechanical Properties

#### 3.3.1. Microhardness

**Figure 16:**
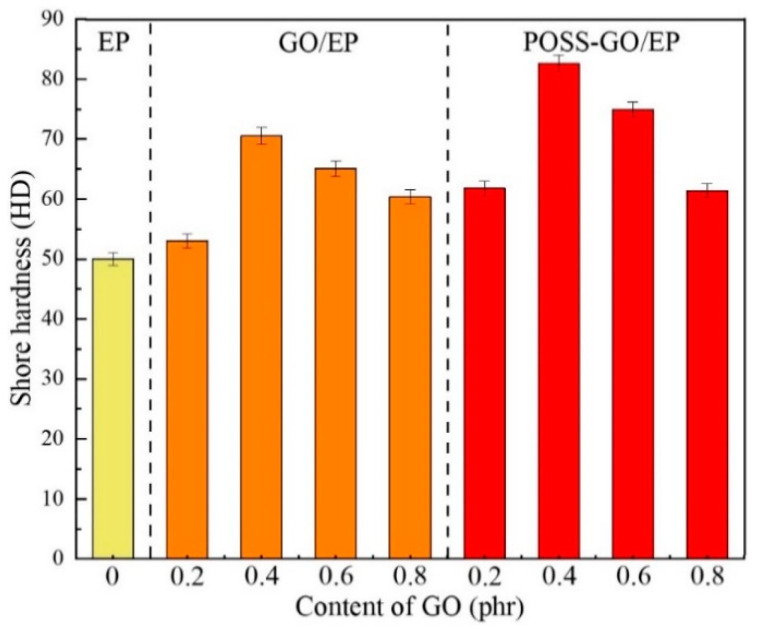
Microhardness of EP, GO/EP, and POSS-GO/EP samples.

Hardness represents the ability of a material to resist local plastic deformation and is one of the important indexes for characterizing the mechanical properties of materials. Figure 16 shows the microhardness test results of EP, GO/EP, and POSS-GO/EP samples. It can be seen that the microhardness of GO/EP and POSS-GO/EP is significantly higher than that of pure EP, and the microhardness of GO/EP and POSS-GO/EP first increases and then decreases with the increase in GO content. Among them, the average microhardness of 0.4 phr POSS-GO/EP reaches the maximum value of about 82.6 HD, which is nearly 65.2% and 17.2% higher than that of pure EP (50 HD) and 0.4 phr GO/EP (70.5 HD), respectively.

Compared with EP, the hardness of GO/EP is significantly increased due to the intrinsic high strength (75 GPa) of GO nanosheets, so their incorporation can increase the rigidity of the molecular chain of nanocomposites. When subjected to external forces, the uniformly dispersed GO nanosheets in the EP matrix can bear part of the load, thereby enhancing the composite material’s ability to resist plastic deformation or damage and improving its microhardness. After functionalization, POSS-GO/EP shows a higher hardness than EP and GO/EP. Particularly, 0.4 phr POSS-GO/EP exhibits the maximum hardness. This is because an appropriate amount of non-covalent functionalized POSS-GO is more evenly dispersed in the EP matrix. Moreover, the meshing effect between EP and POSS-GO is stronger, and the interface binding strength is higher, so POSS-GO can bear the load more effectively. Therefore, the strengthening effect of POSS-GO is more significant.

However, if the content of GO and POSS-GO continues to increase (>0.4 phr), it can inevitably lead to the agglomeration and uneven distribution of GO nanosheets in the matrix, which weaken the rigidity of the molecular chain, deteriorating its bearing capacity and strengthening effects. Therefore, an excessive content of GO and POSS-GO (>0.4 phr) can reduce the microhardness of GO/EP and POSS-GO/EP composites.

#### 3.3.2. Fracture Toughness

**Figure 17:**
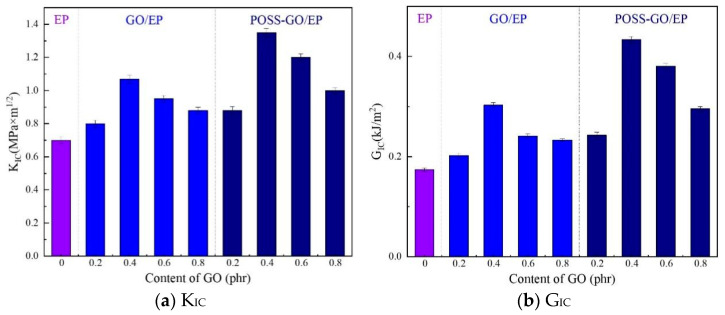
K_IC_ and G_IC_ of pure EP, GO/EP, and POSS-GO/EP.

Fracture toughness is an important mechanical index for characterizing the material’s resistance to the generation and unstable propagation of internal cracks. It evaluates the ability of materials to resist brittle fracture. The K_IC_ and G_IC_ test results of pure EP, GO/EP, and POSS-GO/EP are shown in Figure 17 (where the Young’s modulus data used in G_IC_ calculation are listed in Section 3.3.3). It can be seen that compared with pure EP, the introduction of GO and POSS-GO can effectively improve the K_IC_ and G_IC_ of EP, leading to a toughening effect, and both show a trend of first increasing and then decreasing with the increase in GO content. Among them, the toughening effect of non-covalent functionalized POSS-GO is better than that of GO. In particular, when the POSS-GO content is 0.4 phr, the K_IC_ and G_IC_ of POSS-GO/EP reach the maximum values of 1.35 MPa·m^1/2^ and 0.43 kJ/m^2^, respectively, which are nearly 93% and 153% higher than those of pure EP (0.7 MPa·m^1/2^ and 0.17 kJ/m^2^) and 26.2% and 43.2% higher than those of unmodified 0.4 phr GO/EP (1.07 MPa·m^1/2^ and 0.30 kJ/m^2^), indicating the best toughening effect. This can be attributed to the following reasons: (1) when an appropriate amount of POSS-GO (0.4 phr) is used to toughen EP, it not only ensures a sufficiently high content of POSS-GO in the composite material but also facilitates its uniform distribution in the EP matrix, effectively blocking the propagation of microcracks inside the composite material and enabling the rearrangement of silver lines, shear bands, and molecular chains, resulting in better toughness; (2) the cage-like skeleton structure of the POSS coating layer on POSS-GO is elastic, and its elasticity is similar to that of rubber, so it can play the role of a “marble” [38,39] and effectively absorb energy. The above two effects synergistically improve the strength and toughness of the cross-linking network of EP. Therefore, POSS-GO has a stronger toughening effect on the EP matrix, and the fracture toughness of POSS-GO/EP is significantly improved.

In addition, the fracture toughness of the material can be assessed by calculating the maximum critical crack size ($a_{c}$). According to Griffith’s formula (Formula (5)) [40],

(5) $$K_{IC}=Y\sigma_{b}\sqrt{a_{c}/2}$$

where $K_{IC}$ is the fracture toughness of the material, MPa·m^1/2^; Y is the form factor; $\sigma_{b}$ is the tensile strength value, MPa; and $a_{c}$ is the maximum critical crack size, m. Here, the form factor of composite material $Y=\sqrt{\pi }$, which can be substituted into Equation (5) to obtain

(6) $$a_{c}=2K_{IC}^{2}/\pi \sigma_{b}^{2}$$

**Figure 18:**
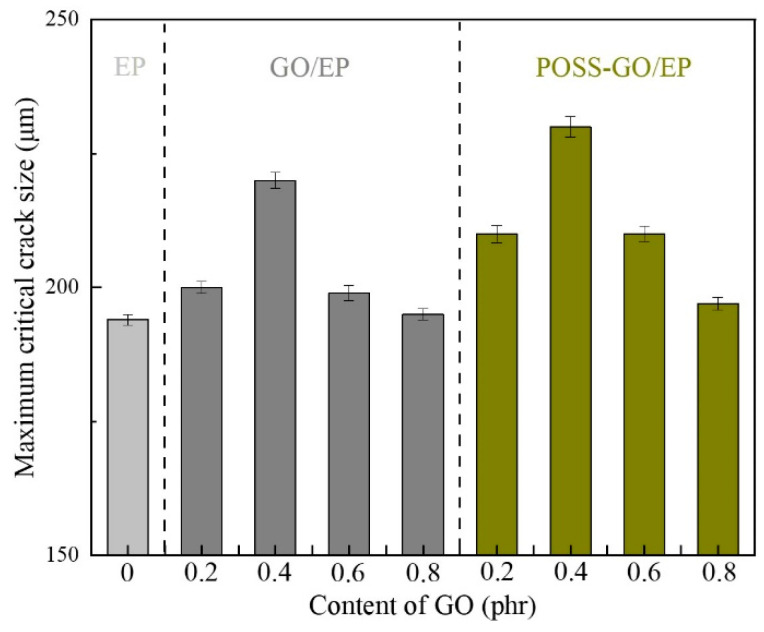
Maximum critical crack size of pure EP, GO/EP, and POSS-GO/EP samples.

Thus, the maximum allowable critical crack size $a_{c}$ in the composite can be calculated by Equation (6) based on its fracture toughness $K_{IC}$ and tensile strength $\sigma_{b}$, and the calculation results are shown in Figure 18 (the tensile strength data are shown in Section 3.3.3).

It can be seen that the maximum critical crack size of pure EP is the smallest at nearly 194μm. Both GO and POSS-GO can increase the maximum critical crack size of EP, and their enhancement effects first increase and then decrease with the increase in GO and POSS-GO content, among which non-covalent functionalized POSS-GO provides a better enhancement effect. When the content of POSS-GO is 0.4 phr, the maximum critical crack size of POSS-GO/EP reaches the maximum value of 230 μm, nearly 19% higher than that of pure EP (194 μm) and 4.5% higher than that of non-functionalized 0.4 phr GO/EP (220 μm). This is because the non-covalent functionalized POSS-GO can help to release the concentrated internal stress near the crack tip inside the material and enhance the stress field intensity near the crack tip, thereby increasing the maximum critical crack size of the material. However, if the content of POSS-GO continues to increase (0.6–0.8 phr), the strengthening effect of POSS-GO is weakened due to the deterioration of dispersion caused by agglomeration, so the maximum critical crack size decreases.

#### 3.3.3. Tensile Properties

**Figure 19:**
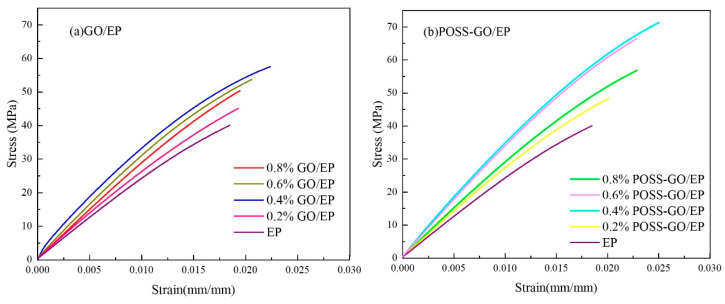
Stress–strain curves of GO/EP and POSS-GO/EP samples with different compositions: (**a**) GO/EP; (**b**) POSS-GO/EP.

**Figure 20:**
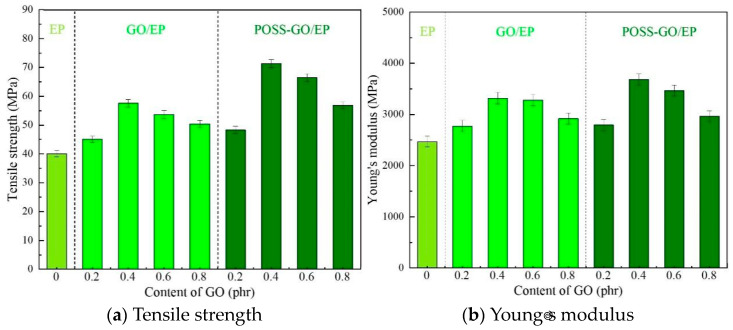
Tensile strength and Young’s modulus of GO/EP and POSS-GO/EP with different compositions.

Figure 19 shows the tensile stress–strain curves of EP, GO/EP, and POSS-GO/EP samples with different compositions, and the corresponding test results of tensile strength $\sigma_{b}$ and Young’s modulus E are shown in Figure 20.

It is clear that the stress–strain curve of pure EP exhibits brittle fracture characteristics. After the addition of GO nanosheets with different contents, the intrinsic brittle fracture characteristics of GO/EP and POSS-GO/EP do not change, but the elongation after fracture is improved. At the same time, the experimental data in Figure 20 show that the ultimate tensile strength $\sigma_{b}$ and Young’s modulus E of GO/EP and POSS-GO/EP epoxy composites are significantly improved when different contents of GO nanosheets are added. Moreover, with the increase in GO and POSS-GO content from 0.2 phr to 0.8 phr, the tensile strength $\sigma_{b}$ and Young’s modulus E of GO/EP and POSS-GO/EP first increase and then decrease. Specifically, POSS-GO/EP exhibits superior mechanical properties (Figure 20), and when the amount of POSS-GO is 0.4 phr, the ultimate tensile strength $\sigma_{b}$ and Young’s modulus E of POSS-GO/EP reach the maximum value (71.38 MPa and 3683 MPa, respectively), which are nearly 78% and 49% higher than those of pure EP (40.08 MPa and 2469 MPa, respectively) and 24% and 11% higher than those of unmodified 0.4 phr GO/EP (57.6 MPa and 3317 MPa, respectively). This indicates that the non-covalent functionalization of GO (POSS-GO) effectively enhances the performance of EP. However, as the content of POSS-GO continues to increase (0.6–0.8 phr), the accumulation and agglomeration of some GO nanosheets occur, leading to a decrease in the degree of dispersion and deteriorating the strengthening effect. Therefore, the tensile strength $\sigma_{b}$ and Young’s modulus E of the composites both tend to decrease.

Based on the above analysis, the enhancement effect of POSS-GO may be ascribed to the following reasons: (1) After functionalization, a large number of benzene rings at the end of the phenyl cage-like skeleton structure are adsorbed on the graphite structure in the GO lamellae through strong π–π coupling, so POSS is firmly coated on the GO surface and embedded between the GO sheet layers. Owing to the steric hindrance effect of the POSS cage-like skeleton, nanomaterials with lamellar structure and larger spacing are formed (as shown in Figure 4). Thus, the agglomeration of GO is inhibited and its dispersion performance in the EP matrix is effectively improved (Figure 15b). Therefore, when POSS-GO/EP composites are subjected to external loads, a large number of POSS-GO uniformly dispersed in EP matrix can bear greater external load due to its excellent mechanical properties, effectively enhancing the performance of EP. At the same time, the uniformly distributed POSS-GO nanosheets can efficiently hinder the molecular chain movement and dislocation of the matrix and stop, redirect, or branch the microcracks generated inside the composite material, effectively suppressing the propagation of microcracks and improving the deformation resistance of the composite material. Therefore, POSS-GO significantly improves the tensile strength $\sigma_{b}$ and Young’s modulus E of the composites. (2) After POSS is modified onto the GO surface, the surface becomes rougher (Figure 13b), and a stronger mechanical interlocking effect is generated at the interface of POSS-GO and the EP matrix, which improves the interfacial force between POSS-GO and the EP matrix as well as the stress transfer efficiency. The load-bearing capacity of the POSS-GO-reinforced phase in the composites can also be utilized to the maximum extent. In addition, it has been reported that the ultimate tensile strength $\sigma_{b}$ and Young’s modulus E of nanocomposites increases with the increase in the dispersion degree of the nano-reinforced phase [41,42,43], which is consistent with the findings of this study.

The Young’s modulus E of nanomaterial-reinforced composites can be predicted by the Halpin–Tsai equation. In order to predict the Young’s modulus of GO/EP and POSS-GO/EP, GO nanosheets are modeled as rectangular cross-sectional fiber reinforcement with width a, length b, and thickness c. The equation based on the Halpin–Tsai model is as follows [44,45,46]:

(7) $$\alpha =\frac{E_{C}}{E_{EP}}=\frac{3}{8}\left(\frac{1+\gamma \eta_{L}V_{GO}}{1-\eta_{L}V_{GO}}\right)+\frac{5}{8}(\frac{1+2\eta_{W}V_{GO}}{1-\eta_{W}V_{GO}})$$

(8) $$\eta_{L}=\frac{\beta -1}{\beta +\gamma }$$

(9) $$\eta_{W}=\frac{\beta -1}{\beta +2}$$

(10) $$\beta =\frac{E_{GO}}{E_{EP}}$$

(11) $$\gamma =\frac{a+b}{c}$$

Substitute Formula (8)–(11) into Formula (7) to obtain Formula (12):

(12) $$\alpha =\frac{E_{C}}{E_{EP}}=\frac{3}{8}\left(\frac{1+\left(\frac{a+b}{c}\right)\frac{\left(\frac{E_{GO}}{E_{EP}}\right)-1}{\left(\frac{E_{GO}}{E_{EP}}\right)+\left(\frac{a+b}{c}\right)}V_{GO}}{1-\frac{\left(\frac{E_{GO}}{E_{EP}}\right)-1}{\left(\frac{E_{GO}}{E_{EP}}\right)+\left(\frac{a+b}{c}\right)}V_{GO}}\right)+\frac{5}{8}(\frac{1+2\frac{\left(\frac{E_{GO}}{E_{EP}}\right)-1}{\left(\frac{E_{GO}}{E_{EP}}\right)+2}V_{GO}}{1-\frac{\left(\frac{E_{GO}}{E_{EP}}\right)-1}{\left(\frac{E_{GO}}{E_{EP}}\right)+2}V_{GO}})$$

In the above formula, $E_{C}$ is the Young’s modulus of graphene oxide-reinforced epoxy resin composites, and its value is based on the experimental results in the paper; $E_{EP}$ is the Young’s modulus of pure epoxy resin, and the $E_{EP}$ given by the manufacturer is 3.10 GPa; $E_{GO}$ is the Young’s modulus of GO, and the $E_{GO}$ given by the manufacturer is 1 TPa; $V_{GO}$ is the volume fraction of GO, and its value can be calculated by converting the mass fraction of GO into volume fraction from the density of GO ($\rho_{GO}=2.25g/{cm}^{3}$) and the density of pure epoxy resin ($\rho_{EP}=1.15\;g/{cm}^{3}$). The relevant sizes of GO nanosheets are estimated based on TEM image, and the average a, b, and c values are about 5 μm, 3 μm, and 4 nm, respectively.

**Figure 21:**
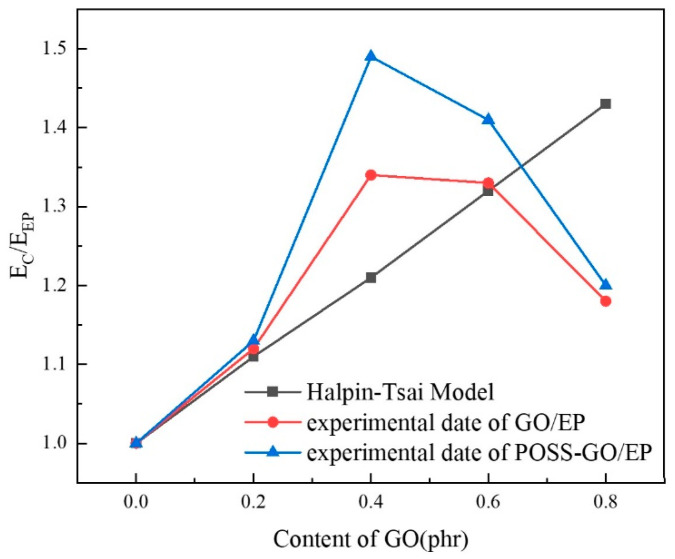
Comparison between the predicted model and experimental results of GO/EP and POSS-GO/EP Young’s moduli.

The comparison results of the theoretical predicted values and experimental measured values of the GO/EP and POSS-GO/EP Young’s moduli are shown in Figure 21. It can be seen that when the content of GO and POSS-GO is ≤0.2 phr, the theoretical predicted value of Young’s modulus is highly consistent with the experimental measured value. When the content of GO and POSS-GO was 0.4 phr, both the theoretical predicted value and the experimental measured value showed an upward trend. In particular, the measured value was significantly higher than the theoretical predicted value due to the relatively uniform dispersion of GO and POSS-GO in the EP matrix. When the content of GO and POSS-GO exceeds 0.4 phr, the experimental measured value of Young’s modulus begins to decline, and the enhancement effect decreases. This is because the dispersibility of GO and POSS-GO begins to decline at this time but still maintains a certain degree of agreement with the theoretical predicted value. In particular, when the content of GO and POSS-GO reaches 0.8 phr, the experimental measured of Young’s modulus has fallen far below its theoretical predicted value. At the same time, it can be seen that the Young’s modulus experimental measured values of POSS-GO/EP are generally higher than those of GO/EP. The reasons for the above phenomenon can be explained from two aspects. Firstly, after the covalent functionalization of POSS, the dispersion of POSS-GO in the EP matrix was significantly improved compared with GO. Secondly, when the mass fraction of GO and POSS-GO exceeds 0.4 phr, with the gradual increase in GO (or POSS-GO) content, GO (or POSS-GO) is more likely to aggregate, and their dispersibility in the EP matrix will begin to decrease, which easily produces structural defects, resulting in local stress concentration during loading. EP breaks too early, and the strengthening effect of GO (or POSS-GO) cannot be fully realized. Therefore, the mechanical properties of the composite materials are decreased. In conclusion, the Halpin–Tsai model can be used to predict the lower limit value of the Young’s modulus of graphite oxide nanosheet-reinforced epoxy resin composite under the premise of the uniform dispersion of graphite oxide.

### 3.4. Tensile Cross-Sectional Morphology and Reinforcement and Toughening Mechanism

**Figure 22:**
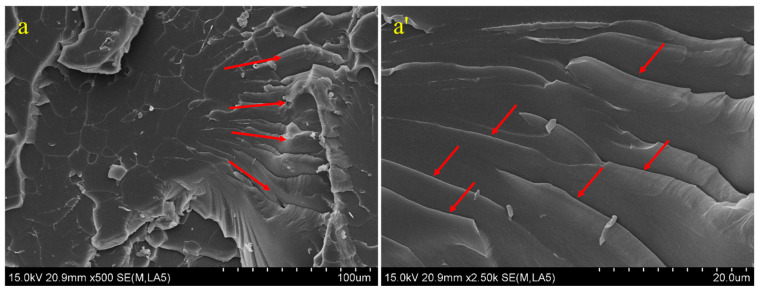

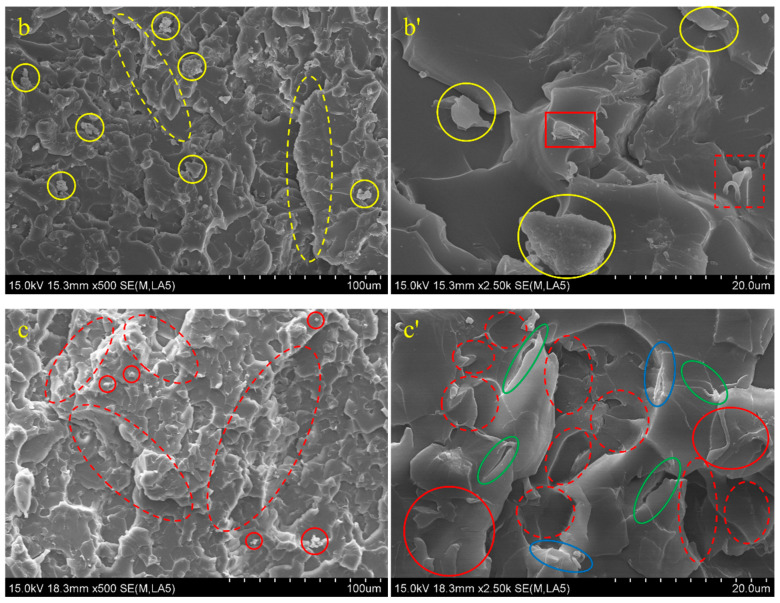
Tensile cross-sectional morphology of epoxy composite materials: (**a**) SEM image of pure EP; (**a’**) high-magnification image of pure EP; (**b**) SEM image of 0.4 phr GO/EP; (**b’**) high-magnification image of 0.4 phr GO/EP; (**c**) SEM image of 0.4 phr POSS-GO/EP; and (**c’**) high-magnification image of 0.4 phr POSS-GO/EP.

The strengthening and failure mechanism of GO-reinforced epoxy composites can be further analyzed by observing the microstructure of the tensile section of composites using SEM. The tensile section microstructure of pure EP, GO/EP, and POSS-GO/EP samples is shown in Figure 22. It can be seen in Figure 22a that the cross-section of pure EP is relatively smooth and flat and presents a typical “riverbed-like” fracture morphology of crack radiation propagation (indicated by red arrow). The cleavage fracture unit on the fracture surface is coarse (up to 100 μm in size). According to the high-magnification image in Figure 22a’, the propagation of cracks is accompanied with a linear expansion, and numerous smooth and regular small ribbons (indicated by red arrow) are formed. The formed microcracks have almost no deflection or bifurcation, and no obvious stress dispersion occurs, which reflects the typical brittle fracture characteristics of pure EP. Therefore, its mechanical properties are low (the tensile strength $\sigma_{b}$ is 40.08 MPa).

Figure 22b shows the tensile cross-sectional morphology of GO/EP. It can be seen that when an appropriate amount of GO is added, the tensile cross-section of GO/EP becomes obviously rougher, presenting a staggered morphology similar to the “fish scale” layer, and there are several “fish scales” with a very small size. This structure can better disperse stress and prevent the formation and propagation of cracks, thereby hindering the occurrence of brittle fracture. At the same time, obvious tortuous fracture traces are observed on the cross-section (the area marked by the yellow dotted circles in Figure 22b), and the size of the cleavage fracture units on the cross-section decreases significantly compared with that of pure EP, where most of these cleavage fracture units have a size near 10 mm. This also indicates that the addition of GO can effectively improve the crack propagation resistance and disrupt the formation of cleavage fracture, thereby improving the tensile properties of EP. In addition, some blocky debris appears on the surface of GO/EP and generates certain plastic deformation (marked by yellow solid circles in Figure 22b,b’), which also suggests that GO/EP can consume more energy, which is conducive to improving its tensile properties. Therefore, the tensile strength of GO/EP (tensile strength $\sigma_{b}$ is 57.6 MPa) is significantly higher than that of pure EP. However, it can be observed from the high-magnification image (Figure 22b’) that there are obvious GO aggregates on the tensile section (red solid box), which is due to the poor dispersion of GO in the EP matrix. At the same time, the peeling phenomenon of GO nanosheets is observed (red dotted box in Figure 22b’), which is because the interfacial bonding between the non-functionalized GO and EP matrix is relatively weak. When subjected to external loads, the weak interfacial bonding between the aggregates of GO nanosheets and EP matrix affects the transfer of interfacial force, leading to the separation of GO and reducing its enhancement effect.

The tensile cross-section of POSS-GO/EP is shown in Figure 22c. It can be seen that compared with the GO/EP tensile fracture surface, the POSS-GO/EP section with an appropriate POSS content also exhibits a staggered rough morphology that is similar to the “fish scale” layer, but the scale layer structure is more obvious; the uneven surface is especially more prominent, and the cross-section exhibits “canine teeth” interleaved stepped fracture traces (the area marked by red dotted circle in Figure 22c). At the same time, the blocky debris on the surface of POSS-GO/EP (marked by red solid circle in Figure 22c) is smaller than that of GO/EP, and the fracture surface has greater roughness. This implies that due to the introduction of POSS-GO, the crack propagation path in the EP resin matrix becomes more complex and longer, and the crack propagation resistance increases, leading to a higher energy absorption, thereby improving the tensile strength of the composite material. According to the high-magnification image of the POSS-GO/EP cross-section in Figure 22c’, many obvious uneven dimple pits are formed on the fracture surface (marked by red dotted circle), and a certain plastic deformation area is observed on the fracture surface (marked by red solid circle). This indicates that the introduction of an appropriate amount of POSS-GO improves the plastic deformation ability of the POSS-GO/EP composite material, and the plastic deformation of the EP matrix becomes more uniform, which leads to more energy consumption [47,48]. In addition, a pull-out phenomenon (blue solid circle in Figure 22c’) and crack pinning phenomenon can be observed on the fracture surface of POSS-GO/EP sample, which lead to crack deflection and bifurcation phenomena (green solid circle in Figure 22c’). This verifies that compared with GO/EP, POSS-GO can more efficiently induce crack deflection and bifurcation in the EP matrix, indicating the enhanced strengthening and toughening effect of POSS-GO. It should be emphasized that no aggregation of GO nanosheets is observed on the fracture surface of POSS-GO/EP, which suggests that the adsorbing of POSS on the GO surface effectively prevents the aggregation of GO lamellae, resulting in a better dispersion of POSS-GO in the EP matrix. Furthermore, no peeled GO nanosheets are observed, indicating that the mechanical meshing between the rougher POSS-GO surface and the EP matrix is stronger and that the interfacial bonding quality is effectively improved, which is more conducive to the transfer of interface stress, synergistically maximizing the reinforcing and toughening effect of POSS-GO. Therefore, POSS-GO/EP exhibits superior mechanical properties (the tensile strength $\sigma_{b}$ is 71.38 MPa).

## 4. Conclusions

Based on the principle of non-covalent functionalization, functionalized POSS-GO was successfully prepared using phenyl POSS as the modifier, and POSS-GO/EP composites were prepared using a solution casting method. The mechanical behavior and toughening mechanism of POSS-GO-reinforced epoxy composites were comprehensively examined. The main results of the study are summarized as follows:

(1) By utilizing the strong π–π coupling between the large number of benzene rings at the end of the phenyl POSS structure and the graphite structure in the GO layer, phenyl POSS was successfully modified onto the surface of GO, achieving non-covalent functionalization of GO (POSS-GO). The introduction of POSS on the surface of GO increased the interlayer spacing of GO, leading to a steric hindrance effect, effectively suppressing the stacking and agglomeration between GO layers and significantly improving the dispersion performance of GO in EP. At the same time, after POSS functionalization, the surface roughness of GO was improved, which was beneficial for enhancing the mechanical meshing between POSS-GO and the EP matrix and for improving the load transfer efficiency, providing full play to the strengthening and toughening effect of POSS-GO.

(2) Compared with pure EP, the mechanical properties of GO/EP and POSS-GO/EP composites were significantly improved. In particular, when an appropriate amount of POSS (0.4 phr) was added, the POSS-GO-reinforced epoxy composites (0.4 phr POSS-GO/EP) showed the best mechanical properties. The microhardness, fracture toughness, tensile strength, and Young’s modulus were 82.6 HD, 1.35 MPa·m^1/2^, 71.38 MPa, and 3683 MPa, respectively, which were 65.2%, 93%, 78%, and 49% higher than those of pure EP and 17.2%, 26.2%, 24%, and 11% higher than those of unmodified GO-reinforced epoxy composites with the same content (0.4 phr GO/EP).

(3) The tensile fracture cross-sectional analysis suggested that POSS-GO was uniformly dispersed in the EP matrix, which could more efficiently induce the deflection and bifurcation of cracks in the EP matrix, causing a certain degree of plastic deformation in the EP matrix for absorbing more external energy. On the other hand, the POSS/EP cross-section was rougher and more uneven, with “canine teeth” interleaved step fracture traces. The crack propagation path was longer and more complex, and the propagation resistance was greater, consuming more energy. At the same time, the mechanical meshing effect between POSS-GO with a rougher surface and the EP matrix was stronger, and the interface bonding quality was effectively improved, which was more conducive to the transfer of interfacial stress. The above synergistic effects promoted the strengthening and toughening effect of POSS-GO, so the resulting POSS-GO/EP composite exhibited excellent mechanical properties.

## Figures and Tables

**Table 1:** Composition of various epoxy composite materials.

Series	EP Matrix (phr)	GO (phr)	POSS-GO (phr)
Neat epoxy	100	-	-
GO/EP composites	99.8	0.2	-
	99.6	0.4	-
	99.4	0.6	-
	99.2	0.8	-
POSS-GO/EP composites	99.8	-	0.2
	99.6	-	0.4
	99.4	-	0.6
	99.2	-	0.8

## Data Availability

Data are contained within the article.
